# Does Information Pattern Affect Risk Perception of Food Safety? A National Survey in China

**DOI:** 10.3390/ijerph15091935

**Published:** 2018-09-05

**Authors:** Guanghua Han, Yihong Liu

**Affiliations:** School of International and Public Affairs, Shanghai Jiao Tong University, Shanghai 200030, China; hanguanghua@sjtu.edu.cn

**Keywords:** risk perception, food safety, information source pattern, public survey

## Abstract

Examining the variances in the assessments of risk, as perceived by residents, facilitates the development of appropriate risk information communication strategies. This paper aims to identify the effects of information source patterns on perceived food safety risks based on demographic factors. A national survey was conducted to examine, by means of multiple regression analysis, the relationship between the public’s perceived risks, demographic factors and information access. The study finds that residents’ preferences for information sources have been empirically proven to significantly affect their perceptions of food safety. We also find that more educated young urban dwellers, as well as those without cohabitation experience, tend to perceive a higher level of risk with respect to food safety. In contrast to our expectations, gender, family income and family size are not significantly correlated with the perception of food safety risk. The findings help to explain residents’ attitudes toward food safety administration and reactions to food hazards in the Chinese context. It is recommended that the governing authorities strengthen their communication capacities using modern communication media and make full use of traditional and face-to-face communications with respect to regulations.

## 1. Introduction

Risk perception has occupied a central position in the agendas of many countries over the last several decades. China’s rapid economic growth and urbanization have had a substantial impact on public risk consciousness. Specifically, the occurrence of food safety incidents in China over the past several years has drawn tremendous attention from the public [1]. 92% of Chinese respondents in a survey believes they expected to soon become a victim of food poisoning and they deem food safety as the second greatest risk in their daily life (after earthquakes) [2]. Food safety has consistently been perceived by the public as an extremely serious problem in China. A Pew Global Attitudes Project reported that, in 2012, 41% of the respondents considered food safety to be an extremely serious problem in China, compared to only 12% who expressed the same attitude in 2008 [3]. In the 4th wave of ABSs (The Asian Barometer Survey (ABSs) traces its history back more than thirty years and aims to conduct cross-national survey focused on democratization in East Asia. The Asian Barometer Project Office (www.asianbarometer.org) is solely responsible for the data distribution.), Chinese residents are asked for their most concerned public issues in their daily life, 13.21% of totally 4068 respondents deem food safety to be the their most concerned issue which is preceded only by issues of economic growth and Primary and secondary education (18.49 and 18.69%, respectively, see Table 1). All the evidence above indicates that the Chinese public perceive lots of anxiety and risk about food safety.

Social scientists and psychologists believe that perceptions of food safety interconnects information communication [4]. The public can access food safety information through a multitude of information sources, such as radio broadcasts, TV reports, newspapers and magazines [5]. The trust in information sources and preference for specific information sources may be key determinants of the public’s perception of risk regarding food safety [6]. Hence, understanding the relationships between the risk perceptions of food safety and the public’s preference for information sources could elicit public approval of governments’ food safety related programs, while optimizing more effective food safety policies [7]. Information source preference should be considered when analyzing the underlying influencing factors of the perceptions of food safety and further actions.

However, research specifically focusing on food safety information sources is limited [7,8,9,10]. This article employs data from a recent representative national survey conducted in 2015 and focuses on communications via various information sources regarding the public perception of food safety risks in China. In addition, we explore the relationship between fundamental demographic characteristics, risk perception and information sources. Meanwhile, incoming states have influence on the behavior and knowledge of residents regarding food consumption [8]. For example, it is found that residents whose family income is less than 1000 US dollars per month are less likely to use cutting boards in preparing meals [9]. As result, the group of family has higher probability to fall in foodborne disease, which may be directly linked with their perception of food safety. An existing study by Chen et al. [10] focuses on a cross-sectional questionnaire survey to workers in three dairy processing plants. The finding demonstrates that workers has low-level knowledge of food safety also presents low satisfactory attitudes about food safety. Thus, we include household economic state as our key control variable in our analysis. The contribution of this study is to provide some understanding of the most reliable paths for communicating food safety information in various social contexts. The conclusion may inspire and guide policymakers as they plan and implement adequate risk communication strategies for food safety policy.

This study’s analysis addresses two major factors. First, perceptions of food safety risk among the general public according to four demographic factors, that is, age, gender, marital status and social identity, are identified with regard to differences in perceptions of risk based on these demographic factors. Second, four types of information sources are combined with the four demographic factors to examine the potential effect of information access on the perception of food safety risk. Finally, underlying explanations are presented and policy insights and recommendations are suggested based on the empirical analysis.

## 2. Theory and Hypotheses

Risk is defined as the set of all destructive consequences that an individual believes possible. As such, risk perception includes all thoughts, beliefs and constructs regarding the characteristics and severity of a risk [11]. In terms of the social construct of risk, the role of information exerts a significant capacity to shape perceptions about risk without direct experience [6]. The level of dependence various populations has on the various sources of information engaged in risk communication. In recent decades, the public’s perception of risk has been examined through psychological experiments and public surveys [4]. Social scientists and psychologists have conducted research into how information impacts and determines perceived risks with respect to food. Information about risks, such as what is reported, how is it reported, to whom and by whom is it reported, is a crucial factor in risk decisions. In this sense, information communication is becoming an essential measure of shaping public’s risk perception and the process of exchanging and sharing information about risk is a purposeful activity. Sharing information about specific risks may enhance interest in given issues, improve public knowledge, change attitudes and behaviors and influence decision-making [12]. In general, information communication can strengthen people’s risk awareness and influence their decisions [13]. In turn, the understanding of the public’s perception of risk improves risk information communication strategies [4]. The sources of information include experts and authorities; traditional media such as radio, television, newspapers and magazines; social media such as the Internet, public agencies and political pressure groups; and informal networks of friends and family. Differences in the structure of the media in various countries may also have important implications for food risk perceptions [14,15].

In China, the government is perceived as the most reliable source of information [10]. Traditional media such as radio, television and newspapers are the most common information sources for individuals seeking risk information [16,17]. Among these sources, television and radio are the primary sources of information regarding food safety [18,19,20]. According to a report from CMMR (China Mainland Marketing Research Company), 97% of residents have access to TV and all television and radio stations are owned by the government for propaganda purposes [21]. As the basic tools of national propaganda, these forms of TV and Radio tend to be more extensive and more positive in their coverage, which can be regarded as self-serving [14]. Magazines and newspaper are the second most preferred media sources. The emergence of media commercialization allows the Chinese media to be more effective in disseminating information and guiding public opinion [22]. Profit-oriented media inevitably offers more negative stories including food safety issues [14]. Since negative information is more attractive than positive [18], the public sometimes prefers to seek reliable information provided by commercial media rather than accessing official media sources [21].

In recent years, the Internet is becoming one of the more common informal ways to reach the population. According to a report from the CMMR (China Mainland Marketing Research Company), approximately 73.2% of the residents in China have access to the Internet. Particularly, when the traditional mass media cannot provide enough information, the public in China is more dependent on the Internet [23]. Additionally, social media also become an effective channel for food safety communication in China [24]. For example, microblogs, which can develop quickly into independent communications platforms have a positive influence on the public’s cognitive learning about food safety information as they are able to bypass censoring of the Chinese government [18,25]. In other Asian countries like Vietnam, people who are female, highly influenced by online relationships and experience difficulty in performing usual activities are more likely to use the Internet to search for information related to food safety [26]. We identify similar observations in our national survey. In particular, female and well-educated (i.e., college-level education or equivalent) respondents and people who prefer online shopping take more time to search for and read information regarding food safety. Meanwhile, the public’s perception of risk is also influenced by other people. Interpersonal exchanges through social networks amplify and attenuate the public’s perception of risk [27]. Particularly, in the absence of access to formal information or when lacking the ability to process complex information, the public may seek one-to-one interactions. Personal contacts and interpersonal trust in China also play important roles in shaping people’s perceptions with respect to risk [28].

The credibility of the information source is an important factor in effective risk communication [29]. Depending on the various levels of credibility regarding the different information sources, we posit that four patterns of information sources have varying effects on individuals’ perceptions regarding food safety risk. First, in consideration of the censoring of news for ideological propaganda in China, the traditional media, including newspapers, magazines, radio and television, is controlled strictly by the government. Thus, the traditional media is more inclined to deliver positive content as it does not support negative content. Second, online media and social media sources, such as the Internet as well as micro-blogs, interpersonal communications and WeChat are relatively free and more flexible in the diffusion of information about safety incidents. Among them, furthermore, the form of media may be more influential than interpersonal communications with respect to risk perceptions and decision-making [30]. Competition from the commercial media and the Web-based media has created a credibility gap problem for the official media [21] as the government often has less public trust than unofficial sources [16]. The public gives more weight to negative information than positive information [14]. Hence, the public, by means of online information sources, is more likely to be affected by media reports about negative information regarding food risk. Thus, we propose the following hypotheses:

**Hypothesis 1** **(H1).** *The various channels of information differently influence people’s perceived food safety risk in China.*

**Hypothesis 1A** **(H1A).** *With respect to traditional media, people who depend on television and radio are likely to express a lower degree of risk with respect to food safety than do people whose media sources include magazines and newspapers.*

**Hypothesis 1B** **(H1B).** *People who depend on traditional media for their information are likely to express a lower degree of risk with respect to food safety than those who use social media as their source of information.*

**Hypothesis 1C** **(H1C).** *People who depend on interpersonal exchange are likely to have a lower degree of risk perception about food safety than those who rely on traditional media and social media.*

In addition to the acceptability of information sources, previous research has found that people’s perceptions of food safety risk vary depending on certain sociodemographic characteristics. Gender, age, education, income, home ownership and geographical region are among the most important characteristics [31,32]. Accordingly, several studies have examined and noted gender differences in risk perception. First, females are usually primary meal planners and caregivers of babies and young children in family in China. Moreover, females are more thoughtful and sensitive in nature. Therefore, women, more so than men, are more vigilant about food safety and tend to express greater concern about the risks associated with technology, the environment and health. Second, older people normally has more experience and knowledge on food-borne diseases. Besides, people pay more attention to health when they are getting old and health problems (e.g., gastritis, digestive disease, cancer etc.) are more likely to arouse their distrust of food safety. Thus, people’s perception of food safety is likely to vary in different life stages. In some previous studies, age is generally found to be positively correlated with risk perception, with older people perceiving greater risk than younger people. Third, educational attainment is an important predictor of public risk perception worldwide. Comparing with the people with less education background, well-educated people usually has more knowledge regarding food induced disease and relatively scientific handing process of food [33]. Therefore, we believe that those with high education have more awareness of food safety risks and tends to exhibit higher levels of risk perception. Actually, income is often taken as an important control variable in empirical studies. In general, income is negatively correlated with risk perception [34]. The high-income group is reasonably more concerned about life quality related issues including food safety and have higher expectations for the safety of food in their daily lives [31]. Thus, we think the low-income people are more apathetic and numb to food safety. Fourth, those with babies and underage children also express higher levels of risk associated with than those who with no children at home. This assumption is straightforward and explainable. Infants and children are more vulnerable to food contaminations, as well as they are less trained and prepared in identifying food risk and food contamination. Additionally, one child policy had been implemented nationally from the early 1980s, most of Chinese family have only one child, Chinese parents are more likely to feel threatened of food safety. Similarly, individuals with marriage or domestic partnership experience express higher levels of food safety risk, since they have experience in preparing meals and are generally becoming more responsible to their lovers. Finally, location, that is, urban versus rural, is likely to influence individuals’ perceptions and concerns about food safety risks. Rural residents put less emphasis on quality but price in food consumption since the family income is normally much lower than that of urban residents. Therefore, urban residents tend to be more concerned about the quality of food than rural citizens because purchasing decisions in rural areas may often be contingent on price consideration [35]. While food safety issue has obtained much attention in contemporary China, systematic examinations of public risk perception of food risk based on national samples are limited. Therefore, this study aims to explore the linkages between perception of food safety and its affecting factors. Socio-demographic characteristics serve mainly as control variables in this study to assess the net influence of cognitive, experiential and socio-cultural factors on risk perception. Based on the review of previous research, we formulate the following hypotheses.

**Hypothesis 2A** **(H2A).** *All else being equal, females and the elderly are expected to perceive higher levels of risk associated with food safety, while residents living in rural areas are expected to perceive lower levels of risk associated with food safety.*

**Hypothesis 2B** **(H2B).** *Individuals with babies or children and those who are married or have domestic partnership experience tend to perceive greater risk with respect to food safety.*

**Hypothesis 2C** **(H2C).** *Individuals with higher incomes and higher levels of education perceive greater risk with respect to food safety.*

## 3. Data and Methods

### 3.1. Data and Sample

The analyses are conducted based on data from the 4th round of Asia Barometer Survey (4th ABSs), which is a tracking survey conducted every three to five years by volunteer institutes and universities in Asia. The ABSs are cross-sectional, face-to-face surveys administered to residents in participating countries. The survey in mainland China was conducted from 1 July 2015 to 6 March 2016, with 6013 eligible samples drawn in the field. The target population covers Chinese rural and urban residents aged 18 and above who have resided in the surveyed communities for no less than one month. The response rate of the survey in mainland China is 67.65%, with 4068 completed, valid interviews. The sampling method uses the “GPS Assisted Area Sampling Method (Landry, F. Pierre and Shen Mingming, “Reaching Migrants in Survey Research: The Use of the Global Positioning System to Reduce Coverage Bias in China”. Political Analysis, 2005, Vol. 13, 1–22. The “GPS Assisted Area Sampling” method was created by the Research Center for Contemporary China at Peking University. Its main advantage is its ability to resolve the complications arising from traditional sampling based on household registration (such as outdated household registration information, excessive empty households, exclusion of migrant population, etc.) as well as the difficulties arising from positioning in area sampling.),” which incorporates population as a measure of size, stratification and multi-stage PPS (Probabilities Proportional to Size). Three rounds of the review were undertaken in every completed questionnaire by the interviewer immediately after leaving the dwelling, by the field supervisor on the same day and later by the data manager in the central office.

### 3.2. Variables and Measurements

Predictor variables include demographic variables as our control variables and behavior variables (e.g., information communication preference) as the predictor variables, while the respondents’ perceptions regarding food safety are the criterion variable.

#### 3.2.1. Perceptions of Food Safety

We asked the respondents to indicate their perceptions regarding the safety of the food they consumed based on their past and present experiences. Their responses were measured using a ten-point Likert scale. Responses ranged from 1 (extremely bad) to 10 (extremely good), with higher values indicating a more positive attitude toward food safety. Because 49.38% of the respondents had a primary-school level of education, it is highly doubtful that they could precisely represent their perceptions of food safety. The respondents’ internal perceptions may not be correctly measured because of semantic differences. Since the respondents’ attitudes were measured by a series of integral numbers between 1 and 10, it is reasonable to deem the respondent as perceiving food as safe if his/her score on food safety is equal to or above 6. Correspondingly, we believe that a respondent’s perception is negative if he/she scores 5 or below on food safety [1,10], indicating the perception that his/her food is unsafe.

#### 3.2.2. Behavior Variables

In this study, we were most interested in identifying the underlying relations between residents’ information source preferences and their perceptions of food safety. In the ABS survey, the respondents are asked to specify the most frequently used channels by which they access domestic and international political news. The options include domestic TV programs, international TV programs, domestic newspapers and magazines, radio broadcasts, domestic websites, international websites, text/Weibo/WeChat messages and face-to-face communications. For our analysis of the different information source preferences, we classify the options in the ABS survey into four categories: TV and radio broadcasts, websites, messages and face-to-face communications. TV and radio broadcasts, which represent more traditional ways to access information, are the most commonly used sources. Therefore, they represent the reference group in our regressions. 

#### 3.2.3. Demographic Variables

Household economic status is measured by the question, “Does your family’s monthly income cover all expenses?” The optional responses are classified into four categories: “Yes and we have some surplus,” “Yes, but we only have a little surplus,” “No, but the deficit is minimal,” and “No and the deficit is substantial.” This variable represents the economic status of the respondents and examines its influence on the perception of food safety. 

The level of education is measured in ascending order with eight categories: 0 = did not complete primary school or below; 1 = primary school; 2 = did not complete middle school; 3 = middle school; 4 = did not complete high school; 5 = high school or technical secondary school; 6 = evening college, technical college, Radio and Television University, correspondence college, self-taught higher education; 7 = full-time undergraduate; 8 = postgraduate or above. In China’s education system, the colleges in category 6 are vocation-based and the students attend on a part-time basis. Accordingly, we combine the items from categories 5 and 6 into one category, high school and equivalent. Similarly, we combine categories 7 and 8 into university graduate or above, categories 3 and 4 into middle school and categories 1 and 2 into primary school. We use university graduate or above as our reference group.

Because families with young children are expected to express concern about food safety, we calculate the existence of underage children in the family based on two questions: “How many people are in your family?” and “How many people over the age of 18 are in your family?” In this way, we determine whether the respondent has at least one underage individual in the household.

As described in above, some individual demographics, such as gender, age and household location, potentially affect residents’ perceptions of food safety risk. Thus, we treat them as explanatory variables in our regression. The questions regarding our measures are presented in Table A1 in the Appendix and statistical details of the variables are presented in Table 2.

Because the respondents’ perception of food safety is an ordinal binary variable, the ordinal logistic regression is employed to empirically identify the influences of the explanatory variables on the respondents’ perception of food safety. Four sets of variables are considered in the regression process: (1) demographic variables, including age, gender and living location; (2) family state, which includes habitation experience and child status; (3) socioeconomic factors, such as education and family income; (4) the criterion variable, that is, the channels the respondents normally access to receive political information. Since each set of variables indicates different aspects of the respondents, we cumulatively explore the effectiveness of the four sets of variables on the criterion variable using four models, namely, M1, M2, M3 and M4 (Table 3).

The analytical findings presented in Table 2 generate several interesting results, some of which corroborate empirical findings in the risk-perception literature. Specifically, three main aspects of the findings are presented.

First, older respondents are more likely to express a positive perception of food safety. For example, given two respondents who are separated in age by one year, the older respondent is 2% more likely to perceive food to be safe. However, there is no significant difference between male and female respondents in their perceptions about food safety. Community size is found to strongly affect the residents’ perceptions of food safety, with residents from large-scale cities generally having more negative attitudes about food safety. For example, the respondents from a large city has only 54.1% probability to perceive safe of food comparing with the respondents from the rural areas.

Second, the educational background of residents significantly affects their perceptions of food safety, where highly educated respondents being more negative about food safety. Specifically, illiterate respondents are 40.37% more likely to believe food is safe than highly educated respondents. Consistent with hypotheses H2C, residents with higher levels of education are proved to perceive greater risks associated with food safety. Meanwhile, residents in a poor economic state are intuitively supposed to focus on basic family expenses [32], such as the education of the child, medical services and so forth. Thus, we assume that family economic state significantly affects the residents’ perception of risk with respect to food safety. However, the empirical results by models M3 and M4 indicate that economic state has no significant influence on an individual’s perception of food safety risk. Contrary to hypotheses H2B, residents with babies or underage children are not statistically found to be different in perception of food safety from those without babies and underage children.

Third, the empirical results verify the difference in the influence of multiple information sources. Specifically, respondents whose preferred information sources are the TV and radio broadcasts do not significantly differ in their perceptions of food safety risk from those respondents who prefer newspapers and magazines. However, the probabilities that respondents who reach news via the Internet (text/Weibo/WeChat) are only 65.05% probability to believe food to be safe comparing with the respondents who obtain their information from the TV are only 65.05% (66.1%). These results indicate that respondents who access information through modern communication channels have a more negative attitude toward food safety. Similarly, respondents who rely on face-to-face communications have more than 95.03% probability of expressing positive attitudes toward food safety.

In summary, the empirical analysis indicates that the older, low-educated respondents who live in small-scale cities, are more likely to have a lower risk perception of food safety. Besides, we also find people who depend on traditional media for their information are likely to express a lower degree of risk with respect to food safety than those who use social media as their source of information. Additionally, people depending on interpersonal information exchanges are most likely to perceive lower risk of food safety than the group who depends on traditional media or social media. Actually, economic state, childhood state and gender have no significant influence on respondents’ risk perception with respect to food safety. In conclusion, Hypotheses H1, H1A, H1B and H1C are fully supported in our empirical study, while H2A, H2B and H2C are unsupported (Table 4).

## 4. Discussion and Implications for Future Practice

During the past few decades, the discrepancy between increasing food safety consciousness and the high frequency of food safety incidents has become quite clear. Accordingly, we examine the influence of different information sources on residents’ perceptions of food safety. Consistent with previous studies, we incorporate demographic variables in our model as control variables and analyze their influences by testing three hypotheses.

### 4.1. Demography Characteristics and the Perception of Food Safety Risk

Consistent with previous studies, we provide certain demographic variables in our model as control variables and propose three hypotheses: H2A, H2B and H2C. However, none of these three hypotheses are fully supported by our analytical results. We find that young, urban and highly educated residents perceive higher levels of food safety risk, while income, gender and having children in the home exert no significant influence on perceptions of food safety risk. These findings are explainable and offer valuable information for government policymakers.

Consistent with several existing studies, age is empirically proven to be positively correlated with perceptions of food safety in this study. Elderly people are generally far more inclined to exhibit low levels of concern regarding food safety than are younger people. One possible explanation is that as people age, they become more patient and tolerant. Furthermore, their personal experiences with food indicate that food safety is far better than it was years ago and is thus less likely to be a threat to their health. Because younger residents are more highly educated and prefer to use modern media in contemporary China, they have more opportunities to learn about food safety scandals and they recognize the drawbacks of the food safety maintenance system, which contributes to their pessimistic perceptions of food safety. In fact, highly educated individuals are empirically found to perceive more risks associated with food safety in both M3 and M4. This observation highlights the direct influence of education on the public’s attitude, with individuals with less education perceiving fewer risks. Thus, education interventions should be offered to improve knowledge and attitudes toward food safety, particularly for less educated individuals [36].

It is interesting that gender is not found to be an influential factor in the perception of food safety in our empirical study, which is contrary to our initial hypothesis. Some surveys reveal that gender does affect concerns about food scandals and that females are more likely than males to perceive higher levels of food safety risk associated with bacterial contamination, additives and pesticide residues. Our empirical results could be a result of the division of labor in Chinese society and in Chinese families. In China, most females are fully employed and both males and females share equally in family duties (e.g., food preparation and cooking, child care, house cleaning, etc.) in the home and at work (e.g., similar workplaces, career tracks and occupations in employment). Thus, similar working and living conditions and experiences between males and females lead to the same levels of vigilance about food safety. In this study, we also find that family income does not significantly influence people’s perceptions with respect to food safety risk. Safety, including food safety, is a universal life requirement of each human being, which explains the non-significant difference among groups with hierarchical family economic statuses.

We examine the influence of family structure on people’s perceptions of food safety risk by introducing two control variables, cohabiting experience and the presence of children in the home. The empirical models M3 and M4 suggest that residents’ habitation experience significantly affects their perception of food safety risk and that the presence of children in the family is highly relevant. This observation is both interesting and understandable. In this study, the residents who cohabit with their significant others begin to adopt marital-type relations and behaviors; thus, they assume new roles and responsibilities in their new family structure compared to the roles that they had when living with their parents or when living as single. In Chinese marital relations, either the husband or both parties make the principal family decisions, including expenditures, investments and insurance plans. Since many food safety scandals have occurred in China in recent years, health and food safety issues have become an important topic in society and a serious concern for families. For example, a newborn child may increase a family’s concern regarding food safety issues but the effectiveness of that concern is not statistically significant. This social characteristic explains the significant influence of cohabiting experiences on residents’ perceptions of food safety risk.

### 4.2. Information Sources and Perceptions of Food Safety Risk

In addition to the demographic variables, the information medium may affect residents’ food safety concerns. We find different perceptions of food risk among groups who rely on various media sources. The results of the quantitative analysis, which are presented in Table 2, support H1B and H1C but do not support H1A. Specifically, we find that those who rely primarily on magazines and newspapers are not significantly different from those who rely on TV. However, people who depend on interpersonal information exchange (i.e., face-to-face communications) perceive less risk with respect to food safety. This finding may result from China’s media regulation policies and the inherent nature of the media in China.

First, TV and radio stations are owned and operated by governments in China and are therefore inclined to deliver positive rather than negative information. However, modern media sources, such as the Internet and Weibo messaging, are commercially owned and more flexible with respect to information sharing and social interactions. Accordingly, information content differs based on the medium sharing the information, with modern media delivering more diversified and negative information.

Second, news spreads faster over modern media, such as the Internet, than it does using traditional media. For example, WeChat is a popular online social media site with more than 980 million users, 61% of whom check information on WeChat more than ten times a day and 55.1% of whom have more than 100 connected friends. Furthermore, information can be shared by a single click on a displayed page. People disseminate more negative than positive information and they tend to disseminate negative information to more recipients over a longer period of time. Thus, a piece of information shared by one person using modern media can be spread rapidly in a short time to an infinitely large number of people.

However, because modern media are less regulated, more negative information and rumors are transmitted. Bad news spreads more quickly and more widely due to the psychology of the users of modern media as well as its natural characteristics. In this sense, it is understandable that residents who rely on modern media sources receive more negative information and hence perceive a higher level of food safety risk. Among the modern media in contemporary China, text, Weibo, blogs and WeChat services request that members provide individual personal information, whereas posts and comment services on the Internet are typically anonymous. It is reasonable to assume that more negative information, as well as vicious comments and rumors, are transmitted over the Internet. As a result, residents who rely on the Internet for information perceive greater risk than do those who rely on text, Weibo, blogs or WeChat.

Finally, people who depend on interpersonal information exchanged through face-to-face encounters experience the lowest degree of risk perception regarding food safety among traditional media and modern media in our empirical study, which may be due to two separate factors. First, most (approximately 83% in our survey) residents who depend primarily on interpersonal information exchanged through face-to-face encounters are either elderly or living in rural areas and have less education. As shown in this study, rural and elderly residents are more likely to perceive fewer food safety risks.

In summary, this study examines the influence of information sharing media on residents’ risk perceptions of food safety in the context of China by introducing residents’ demographic characteristics as control factors. We find that residents who rely on social media perceive a higher level of food safety risk than do those who rely on traditional media. Relying on interpersonal information exchanges leads to the lowest level of risk perception regarding food safety. The empirical findings from the analysis of the national survey data reveal some patterns in respondents’ perceptions of food safety that are potentially helpful in conceiving governmental policies.

### 4.3. Limitations and Future Studies

The limitations of this study are at least twofold. First, we have omitted some variables. Perceived risk of food safety depends not only on different sources but source credibility, content of the message, as well as the amount and quantity of information. It is worth including more variables to explain food safety perceptions from different dimensions. Moreover, psychometric paradigms argue that perceived benefit, trust, negative image and knowledge influence the perceived risk of food. In our future research, we will pay special attention to the variables and endeavor to provide more explanations of residents’ food safety perceptions. Second, exploring influence factors such as information sources in this study is important and contributive but it is also valuable to examine how the public’s perception of food safety affects their behaviors. For example, food safety perception is likely to affect the public’s attitude to government, political participation and social trust, the empirical studies to examine their relations are therefore worthwhile. Thus, investigating the impact of the perception of food safety offers a fertile avenue for future research.

## Figures and Tables

**Table 1:** Chinese respondents’ most concerned issues.

The Most Important Public Issues in Respondent’s Belief	Proportion in All 4068 Respondents
Economic Growth	18.46%
National defense and foreign policy	6.87%
Environmental protection	7.89%
The gap between rich and poor	8.23%
Official corruption	10.77%
Job opportunities	4.99%
Food safety	13.21%
Public health and medical services	10.88%
Primary and secondary education	18.69%

Note: Data from the 4th ABSs.

**Table 2:** Sociodemographic statistics for the sample of respondents.

Sociodemographic Characteristic	Percentages	Sociodemographic Characteristic	Percentages
Perception of Food Safety		Gender	
Safe	49.56%	Male	48.90%
Unsafe	51.44%	Female	51.10%
Age		Educational Attainment	
18–29	16.74%	Literacy	27.49%
30–39	14.07%	Primary school	21.89%
40–49	21.22%	Middle school and Equivalent	25.48%
50–59	19.81%	High school and Equivalent	15.64%
Over 60	28.15%	College and above	9.49%
Underage Children		Habitation Experience	
Have	42.09%	Have	87.8%
Do not have	57.91%	Do not have	12.2%
Location		Preferred Information Source	
Central City	9%	TV	51.2%
Regional City	0.59%	Newspaper/Magazine/Radio	10.63%
Town	25.36%	Broadcast	
Rural	56.04%	Internet	21.96%
Economic State		Text/Weibo/WeiChat Message	11.04%
Huge of Deficit	10.71%	Face-to-Face	5.17%
Some Deficit	23.22%		
Balance	39.88%		
Some Surplus	26.19%		

**Table 3:** Perception of food safety risk with respect to four sets of variables.

Perception of Food Safety	M1	M2	M3	M4
Age	0.020 ***	0.024 ***	0.018 ***	0.015 ***
	(0.002)	(0.002)	(0.003)	(0.003)
Gender (Female)				
Male	0.035	0.042	0.044	0.050
	(0.066)	(0.066)	(0.066)	(0.066)
Location (Rural)				
Town (<0.1 Million)	−0.548 ***	−0.552 ***	−0.451 ***	−0.411 ***
	(0.092)	(0.093)	(0.095)	(0.095)
Regional City (0.1 Million to 1 Million)	−0.851 ***	−0.859 ***	−0.655 ***	−0.600 ***
	(0.113)	(0.114)	(0.118)	(0.119)
Central City (>1 Million)	−1.090 ***	−1.106 ***	−0.822 ***	−0.779 ***
	(0.114)	(0.114)	(0.123)	(0.123)
Habitation Experience (with habitation experience)				
Others		0.302 **	0.434 ***	0.464 ***
		(0.125)	(0.128)	(0.129)
Underage Children (have)				
No Children		−0.090	−0.065	−0.060
		(0.07)	(0.071)	(0.071)
Education (College and Above)				
High School and Equivalent			0.372 ***	0.314 **
			(0.131)	(0.132)
Middle School and Equivalent			0.653 ***	0.556 ***
			(0.131)	(0.133)
Primary School			0.756 ***	0.652 ***
			(0.139)	(0.142)
Literacy			1.001 ***	0.877 ***
			(0.149)	(0.152)
Family Economic State (poorest)				
Poor			−0.125	−0.121
			(0.137)	(0.137)
Fair			0.103	0.132
			(0.13)	(0.13)
Good			0.077	0.121
			(0.137)	(0.137)
Information (TV)				
Newspaper/Magazine/Radio Broadcast				0.050
				(0.159)
Internet				−0.430 ***
				(0.112)
Text /Weibo/WeChat Message				−0.414 ***
				(0.133)
Face-to-Face				0.668 ***
				(0.188)
Constant (Safe)				
Unsafe	1.929 ***	1.793 ***	1.321 ***	1.609 ***
	(0.127)	(0.141)	(0.210)	(0.222)
R2	0.079	0.091	0.187	0.254
Adjusted R2	0.082	0.097	0.201	0.278
N	3568	3525	3476	3103

Note: standard errors in parentheses; *** *p* ≤ 0.01;** *p* ≤ 0.05; two-tailed test.

**Table 4:** Results in Hypotheses Tests.

Hypotheses	Testify Results
H1, H1A, H1B, H1C	supported
H2A, H2B, H2C	partial supported

## Appendix A

**Table A1:** Main measures and questions.

Measures	Questions	Selections
Perceptions of food safety	How would you rate the overall food safety condition of our country today?	(extremely bad) 0, 1, 2, 3, 4, 5, 6, 7, 8, 10 (extremely good)
Household economic status	Does your family’s monthly income cover all expenses?	1. yes and we have some surplus
		2. yes, but we only have a little surplus
		3. no, but the deficit is minimal
		4. no and the deficit is substantial.”
Education level	What is your highest level of education?	0. did not complete primary school or below
		1. primary school
		2. did not complete middle school
		3. middle school
		4. did not complete high school
		5. high school or technical secondary school
		6. evening college, technical college, Radio and Television University, correspondence college, self -taught higher education
		7. full-time undergraduate
		8. postgraduate or above
Existence of underage children	How many people are in your family? How many people over the age of 18 are in your family?	Interviewers fill in the blankets.
Age	What is your birth year?	Interviewers fill the years in the blankets and converted it to actual age.
Gender	No questions here	Interviewers fill in the blankets.
Habitation Experience	What is your marital status?	1. single/Never married
		2. married
		3. living-in as married
		4. widowed
		5. separated/married but separated/not living with legal spouse
		6. divorced
Most used information channels	Which are the most commonly channels by which you usually access domestic and international political news.	1. TV programs
		2. international TV programs
		3. domestic newspapers and magazines
		4. radio broadcasts
		5. domestic websites
		6. international websites
		7. text/Weibo/WeChat messages and face-to-face communications.
